# Toner Lectures

**Published:** 1873-05

**Authors:** 


***Toner Lectures.***

A series of lectures has been instituted at Washington under the "Toner Fund to encourage the discovery of new truths for the advancement of Medicine." The trustees of the fund are the following distinguished gentlemen:

JOSEPH HENRY, LL.D., Secretary Smithsonian Institution.
JOSEPH K. BARNES, M.D., Surgeon General U. S. A.
J. C. PALMER, M.D., Surgeon General U. S. N.
JAMES E. MORGAN, M.D., President Medical Society, D. C.
J. M. TONER, M.D.

We take pleasure in acknowledging receipt of an invitation to attend, which we should most cordially accept if in our power. This is a most encouraging movement in the right direction, evincing a tendency to rise above questions of medical etiquette, points of order, and dubious disquisitions on "elevation of the profession" by resolution.

Another such a course should be inaugurated in Chicago, the metropolis of the Northwest. Let both the public and the profession know that earnest work, with rich reward of discovery, is going on in the domain of medical science. As illustrative of what is being accomplished at the capital we extract the following from the Washington Republican of the 19th ult.:

" *The Nervous Force. Dr. Brown-Sequard's Lecture Last Night.* —Dr. Brown-Sequard's lecture on 'The Nervous Force'—the second of the Toner course—was delivered last evening at Marini's Hall, before an audience remarkable alike for quantity and quality. The doctor's world-wide fame appeared, notwithstanding the threatening aspect of the weather, to have drawn out the whole army of Washington scientists. In every part of the room might be seen the well-known face of some gentleman who has won local distinction by his persistent efforts in extending the bounds of human knowledge, or storing up in his mind the result of previous investigation. A few *dilletanti* hung about the skirts of the crowd, and row upon row of doctors in solid phalanx filled the body of the hall. Even quite a bevy of ladies were present, and an equal number of blushes at certain parts of the lecture.

" Professor Henry introduced the lecturer with a few laudatory remarks, referring incidentally to the recent great advance of the cause of science in this country, the liberal donations of Congress, and the bright prospects of the Smithsonian Institution.

" Dr. Sequard's remarks were concise, and replete with valuable facts. No inquiry was attempted into the occult causes and nature of the subtle power which caused the strange phenomena that he described. Once or twice some telling anecdote or bit of illustration was introduced, provoking hearty peals of laughter.

" The speaker, after a few preliminary remarks on the subject of his lecture, said that the variety of phenomena in the nervous system is much greater than is generally supposed, even by medical men. All sorts of skin eruptions are more or less traceable to nervous disorder. Experiments in animals had shown that the eruption followed immediately after exciting certain nerve centres. It was only within the last ten years that it has been discovered that a bedsore was due, not to pressure of the bed, but to spinal lesion, or spinal irritation; when, for instance, an irritation in the spinal column was produced, a bedsore appeared. They often come on parts subjected to no pressure whatever.

" Elephantiasis—a disease peculiar to South American countries —may be caused by an affection of the nerves by tumor. Bones are injured by nervous influences, as are joints, the former not only diminishing in size, but their marrow wasting. Pain in the joints, is caused by an irritation at the base of the brain.

" The lecturer referred to various physiological effects caused by an irritation at the base of the brain, giving among many instances the following: The knee in one night might be made twice its usual size. Immediate injury to the lungs might be produced. A number of white corpuscles could be made to appear in the blood; indeed, the brain itself should be considered as a part of the nervous system. The irritation arising from severed nerves sometimes caused an injury to the spinal cord. A lesion of the spinal cord might change the structure of the brain.

"Disease of any part of the brain does not necessarily arise directly from injuries to that particular part, but may proceed from some other part of the nervous system. An irritation starting from the cerebellum, on the left side of the head, often causes loss of sight of the right eye or *vice versa*.

"An injection of cold water in the ear will make a person stagger or trot round in a circle like a horse in a circus. The Duke of Wellington, like many other noblemen, was stupid enough one time to consult a quack, who injected nitrate of silver in his ear, and set the duke rotating faster than he (the lecturer) could make his arm go. [Laughter.] The lecturer here read an extract descriptive of a high-seasoned dish, of which Frederick the Great one time partook. Not satisfied with this mess of garlic and spiced meats, he ate directly afterwards a veal pie so hot that it seemed to have been baked in hell. He was seized with convulsions. Perhaps it would have a different effect on the speaker as he was not a great man. [Laughter.]

"A great variety of effects were produced by apparently the same cause, as in the case of persons in a perspiration coming out of a theatre. Some took cold in the head, lungs, throat, and so on. In each nervous system there are parts which are easier to excite than others. Exciting certain nerves will contract blood vessels, and produce attraction of the blood by tissues. When the nerve running to the heart is galvanized, the action of the heart is stopped. Indeed, whenever we breathe we produce a decrease of action of the heart. The exchanges between tissue and blood are stopped by certain forces of the nervous system. The loss of consciousness in epilepsy is caused by irritation of the *medulla oblongata*.

"Stick a pin in your spinal column and it will produce complete paralysis. Coughing can be stopped by distending one's nostrils with the finger and thumb. A pressure of the finger upon certain parts of the face will stop sneezing, and by pulling down the sufferer's big toe convulsions may be stopped.

"The lecturer, after giving several examples of this character concluded his address by thanking the audience for its attendance and attention."

The Chronicle says: "If the doctor could have had six hours to present what he was forced to crowd into one, he would have suited himself and his audience much better, but no one could do more than he did in the same time."

